# The impact of empathy, sensation seeking, anxiety, uncertainty, and mindfulness on the intercultural communication in China during the COVID-19

**DOI:** 10.3389/fpubh.2023.1223215

**Published:** 2023-07-13

**Authors:** Muhammad Umar Nadeem, Steve J. Kulich, Anastassia Zabrodskaja, Ijaz Hussain Bokhari

**Affiliations:** ^1^SISU Intercultural Institute (SII), Shanghai International Studies University (SISU), Shanghai, China; ^2^Baltic Film, Media and Arts School, Tallinn University, Tallinn, Harju County, Estonia; ^3^School of Commerce and Accountancy, University of Management and Technology (UMT), Lahore, Punjab, Pakistan

**Keywords:** COVID-19, mindfulness, anxiety, uncertainty, empathy, sensation, China

## Abstract

**Objective:**

This study seeks to explore factors that have shaped the intercultural communication effectiveness (ICE) of international students (IS) during the COVID-19 pandemic. Theoretical predictions of anxiety uncertainty management (AUM) are considered to assess the ICE of IS who stayed in China throughout the COVID-19 pandemic. The prime causal factors of AUM theory (anxiety, uncertainty, and mindfulness) are included with empathy and sensation, seeking to examine their impact on ICE among IS in China.

**Methods:**

A quantitative research design was designed to survey IS via convenience samples from across China with a total of 261 IS from 42 different cultural backgrounds responding to invitations to participate in a Chinese–English survey. Well-established measurement tools were adopted to measure empathy (Cultural Empathy scale), sensation seeking (Brief Sensation Seeking Scale), anxiety (Intercultural Anxiety scale), uncertainty (Intercultural Uncertainty scale), mindfulness (Cognitive and Affective Mindfulness Scale-Revised), and ICE (Perceived Effectiveness of Communication scale).

**Findings:**

The findings revealed that anxiety (*t* = −3.61, *p* < 0.05) and uncertainty (*t* = −2.51, *p* < 0.05) had a negative impact on ICE. However, mindfulness (*t* = 3.93, *p* < 0.05), empathy (*t* = 3.60, *p* < 0.05), and sensation seeking (*t* = 7.93, *p* < 0.05) had a positive influence on ICE. Furthermore, the moderating effect of mindfulness is affirmed in this study.

**Conclusion:**

This study has reconfirmed the theoretical reasonings and applicability of AUM theory with the addition of empathy and sensation seeking by IS in the cultural context of China during the COVID-19 pandemic.

## 1. Introduction

China has become a key destination for international students (IS) from all over the world, and thus a key location for re-examining intercultural communication experiences, interactions, and research (1, 2). The Chinese Ministry of Education (MOE) both planned for and succeeded in recruiting 500,000 students by 2020 (3) to become the top talent hub in Asia (4). The government provided different scholarships to attract such students, which both created an improving global image and expanded circles of international relationships (5). Even though China has been becoming one of the best destinations for IS, they still encounter issues regarding a new culture. The literature confirms that studying in a new culture brings a wide range of sociocultural, psychological, and academic challenges (6). Studies on IS in China have duly focused on aspects of their stress, coping, adaptation mechanisms (7, 8), culture learning strategies (9), interactions, and competence development (10).

Such a focus became even more critical for current and future IS after the COVID-19 pandemic began. A wide range of studies on IS who were living and stayed on in China during the COVID-19 pandemic reported their psychological issues, such as higher levels of loneliness, fear, stress, anxiety (11), depression (12), and psychological distress (13). Though previous research has explored both psychological and adjustment dynamics of IS in interactions (14), their intercultural communication effectiveness (ICE) has been largely neglected, and thus not explored during the various pandemic lockdowns. Few studies have focused on the psychology or communication effects of dealing with anxiety or uncertainty (15), and even less on how these can be managed from the lens of anxiety uncertainty management (AUM) theory in intercultural communication contexts (16–18). Therefore, an assessment linking the predictors of ICE through the perspective of AUM is needed to understand how these longer-term IS staying in China managed psychologically during COVID-19 pandemic (19).

AUM theory revolves around examining the linkages among anxiety, uncertainty, and mindfulness to achieve an understanding of how people in intercultural contexts achieve ICE (20, 21). The theory has been applied in numerous cultural contexts such as the United States (20), Australia (22), China (15), Malaysia (6), and Pakistan (18). Researchers have extended the AUM framework with potential variables arising from their respective disciplines, including health care, public relations, digital media, psychology, and management (23–27). These lines of research have significantly contributed to confirming AUM theory as well as its applicability and extension to diverse settings (18) in typical intercultural communication contexts. However, the exigencies imposed on people by the COVID-19 situation potentially imposed new stressors, with unexpected levels of uncertainty and anxiety that likely affected communication attitudes or behaviors among people from different cultures. Therefore, the current study aims to revisit AUM theory by introducing several more recently noted variables that might affect IS in the cultural context of China during the COVID-19 pandemic.

Intercultural research adopts two approaches, with some scholars predominantly adopting a culture-specific view (an emic, local, or indigenous oriented approach) to explore descriptive intercultural communication phenomena among two cultures (28, 29). In contrast, the culture-general view (an etic, broadly comparable, or generalized approach) argues that the culture-specific view cannot be universally applicable due to the biases or limitations of specific cultures (30, 31). The culture-general view targets common traits or features of more than two cultures to improve its validity and generalizability to many cultures (32). It can thus be applied to the ability of a person to communicate effectively across different cultures (33). Previous efforts to identify and explore culture-general factors have added valuable insights to the literature of intercultural communication (34, 35), developing important, widely tested models affecting ICE. AUM is one of the most widely adopted models, but unfortunately has been minimally investigated in China, and specifically not for IS. This suggests the importance of testing the key factors of AUM among IS in China, who not only come from different cultures, but who must find ways to be more effective in intercultural interactions with a culturally diverse population (and even more during COVID-19).

**Figure 1:**
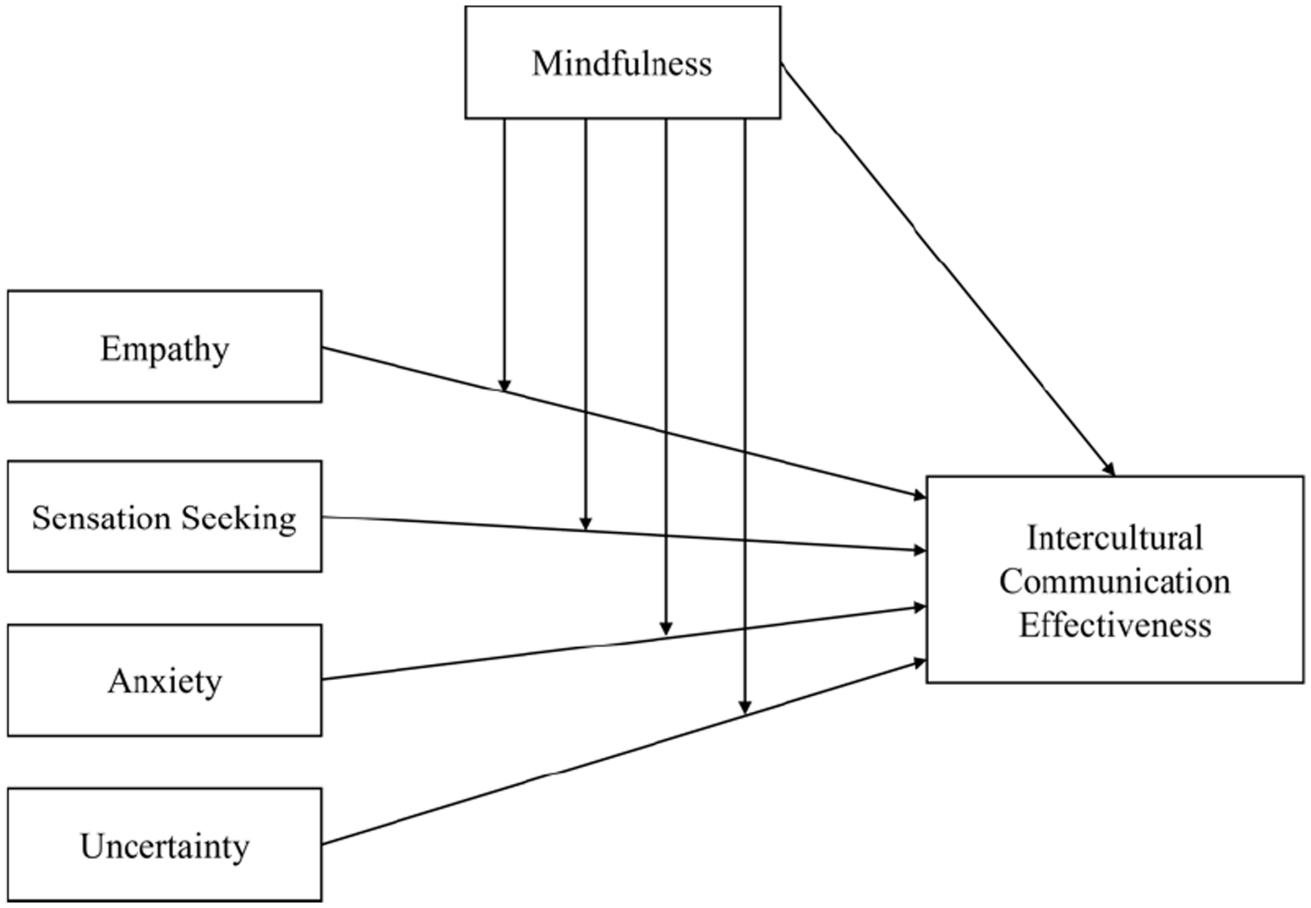
Extended theoretical framework of the AUM theory.

The present study aims to extend AUM theory with two promising indicators (empathy and sensation seeking) incorporated into the integrated model of intercultural communication competence (IMICC), that have direct relevance to intercultural communication (35–37), and which have not yet been either duly considered as additional basic causes of ICE (16, 17, 20, 38) in the AUM theoretical framework, or in the lesser researched cultural context of China. Logically, the AUM factors (anxiety, uncertainty, and mindfulness) as well as the IMICC’s addition of empathy and sensation are psychological dispositions that might help people function in extreme situations. However, the question remains as to how they were in fact used or resorted to by the IS as they sought or struggled to function in extreme situations and find effective ways to communicate interculturally during the COVID-19 constraints in China (see Figure 1). Therefore, the focus of this study is to expand and test AUM theory related to ICE in a new context (China) during the intensely uncertain situation of COVID-19 for the reconfirmation and expansion of its theoretical groundings.

## 2. Methods

A cross-sectional research design utilizing an online survey was adopted for this study. The short 39 item survey questionnaire (requiring approximately 15 min to complete) was linked with a QR code and embedded in an “invitation to participate” poster that was shared on various online and offline platforms to recruit IS who still resided in China. Since the statistics regarding those IS who stayed in China during the pandemic have not yet been officially published, it proved nearly impossible to estimate the exact number of IS still on location during that time. Therefore, we incorporated a non-random sampling technique (convenience sampling) to approach the respondents, even though the incorporated sampling technique might confine the generalizability of the results and the selected samples might not fully represent the actual population. Yet, the study approached IS to reveal their communication behaviors during this intense time.

Since IS statistics have not been released since 2019, the current study has considered the sample-to-variable ratio for the determination of target sample size (39). The adopted technique suggests that a sample-to-variable ratio of 20:1 is preferred, which means a minimum of 120 samples are required for this study (120:6). Since the available pool of IS was limited and most students could not return to China once COVID-19 emerged in January 2020, many of those who initially stayed found ways to return home whenever there were small openings between pandemic lockdowns. With a greatly reduced available sample, it was important to try to attract broader participation among those IS still in China; therefore, the circulated survey included a request that each participant help by sending it to others (utilizing snowball sampling). Overcoming such challenges, the study recruited 261 IS who validly completed the survey, going beyond the minimum requirements of sample size determination.

### 2.1. Measurement tools

All measurements of the variables were done bilingually (English and back-translated Chinese equivalent items) with a Likert scale ranging from 1 to 5 (strongly disagree to strongly agree). The dual language convention was adopted because most IS in China generally have a reasonable command of the English language and they are expected to learn basic Chinese language skills before beginning their academic courses (40). For this reason, we translated the English items into Chinese (and checked equivalence) to facilitate the process and make sure that the meaning of items was clear (no matter which language they preferred or checked).

Standard demographics were also included in the survey, with some additional closed- and open-ended spaces to provide information regarding their age, gender, education, and other basic factors. The key variables of the study included: empathy, measured through the eight item cultural empathy subscale of the Multicultural Personality Questionnaire short form (MPQ-SF) (41); sensation seeking, measured using the eight items of the Brief Sensation Seeking Scale (BSSS) (42); anxiety, measured through the five items of the intercultural anxiety scale (43); uncertainty, measured through the three items of the intercultural uncertainty scale (22); mindfulness, measured through the ten items of the Cognitive and Affective Mindfulness Scale-Revised (CAMS-R) (44); and ICE, measured through five items of the perceived effectiveness of communication scale (45). Table 1 presents all associated details about the variables of this research.

**Table 1:** Variables’ details.

Variable	Item	Minimum	Maximum	Previous Alpha	Present Alpha
Empathy	08	1	5	0.810	0.854
Sensation seeking	08	1	5	0.760	0.896
Anxiety	05	1	5	0.860	0.877
Uncertainty	03	1	5	0.800	0.828
Mindfulness	10	1	5	0.770	0.938
ICE	05	1	5	0.820	0.931

### 2.2. Statistical procedures

All statistical analyses were performed in the Statistical Package for Social Sciences (SPSS) version 23.0 through four different steps. First, descriptive statistics were performed to report the demographic patterns of IS. Second, the reliability and validity of all variables were ensured before executing the final analysis. Third, multivariate regression analysis was performed to report the direct effects of all variables on ICE. Lastly, three different techniques (slopes inspection, interaction effects, and change in R^2^) were adopted to examine the moderating effect. The supportive details of all performed analyses are discussed in the following section.

## 3. Findings

### 3.1. Demography of international students

A wide range of 42 national cultures were represented by the 261 IS studying in public and private higher education institutes in different regions of China, in which the majority of them were from South Korea (*n* = 42), Russia (*n* = 31), Thailand (*n* = 30), and Pakistan (*n* = 17). Approximately 77% of the IS reported having less than 3 years and the remaining 23% had more than 3 years of past international experiences. Both male (*n* = 108) and female (*n* = 151) students participated in the study. Religious orientation was classified into four main categories: Buddhist (*n* = 86), Muslim (*n* = 76), Other (*n* = 58), and Christian (*n* = 41). A total of 72.8% of students were enrolled in Bachelor programs and the remaining 27.2% identified themselves as enrolled in Masters and Doctoral degrees. Regarding age, 190 students were between 16 to 25 and the other 71 students were older than 26. All supportive details of demographic patterns of the participating IS are shown in Table 2.

**Table 2:** Demography of international students.

		Frequency	Percentage
1. What is your Nationality?	Algeria	2	0.8
	Argentina	2	0.8
	Armenia	2	0.8
	Bangladesh	3	1.1
	Bolivia	2	0.8
	Brazil	4	1.5
	Cambodia	7	2.7
	Colombia	3	1.1
	Egypt	6	2.3
	France	2	0.8
	Germany	2	0.8
	Ghana	6	2.3
	Indonesia	2	0.8
	Italy	4	1.5
	Japan	9	3.4
	Kazakhstan	7	2.7
	Lebanon	2	0.8
	Liberia	4	1.5
	Lithuania	2	0.8
	Loas	2	0.8
	Malawi	3	1.1
	Malaysia	2	0.8
	Mongolia	2	0.8
	Morocco	6	2.3
	Myanmar	1	0.4
	Nepal	7	2.7
	Nigeria	2	0.8
	Pakistan	17	6.5
	Russia	31	11.9
	Rwanda	3	1.1
	Senegal	3	1.1
	South Korea	42	16.1
	Spain	6	2.3
	Tanzania	4	1.5
	Thailand	30	11.5
	Uganda	2	0.8
	Ukraine	1	0.4
	Uzbekistan	9	3.4
	Vietnam	9	3.4
	Yemen	2	0.8
	Zambia	4	1.5
	Zimbabwe	2	0.8
2. Previous international experiences?	< 1 year	156	59.8
	2–3 years	44	16.9
	3–4 years	20	7.7
	> 4 years	41	15.7
3. What is your gender?	Female	151	57.9
	Male	108	41.4
	Other	2	0.8
4. What is your religion?	Buddhist	86	33.0
	Muslim	76	29.1
	Other	58	22.2
	Christian	41	15.7
5. What is your education?	Bachelor	190	72.8
	Master	39	14.9
	PhD	24	9.2
	MA/PhD	8	3.1
6. What is your age?	16–25	190	72.8
	26–35	60	23.0
	36–45	8	3.1
	46–55	3	1.1

### 3.2. Results of reliability and validity

The assessment of reliability and validity was ensured before starting the final regression analysis. The indicators and their criteria of assessments are available in and follow the standard literature regarding the evaluation of reliability and validity of the measurement tools of variables. The values of Cronbach Alpha (α > 0.70) were assessed for the evaluation of the reliability of measurement tools. The results showed that the value of α was 0.854 for empathy, 0.896 for sensation seeking, 0.877 for anxiety, 0.828 for uncertainty, 0.938 for mindfulness, and 0.931 for ICE, which were higher than previous research (see Table 1). In terms of validity, confirmatory factor analysis (CFA > 0.50) was performed. CFA results showed that every single item loaded significantly in their respective variable and exceeded the value of 0.50. Table 3 contains the complete details of every item as well as their loading concerning each variable of the current study. The two step assessment results confirm that the data is reliable and valid enough for further analysis.

**Table 3:** Instruments details.

Variable/Item	Loading
**Empathy (M = 3.37, SD = 0.78)**	
1. I pay attention to the emotions of others	0.877
2. I am a good listener	0.812
3. I sense when others get irritated	0.888
4. I enjoy getting to know others profoundly	0.801
5. I enjoy other people’s stories	0.808
6. I notice when someone is in trouble	0.851
7. I sympathize with others	0.832
8. I set others at ease	0.865
**Sensation seeking (M = 3.60, SD = 0.87)**	
1. I would like to explore new places	0.904
2. I get restless when I spend too much time at home	0.914
3. I like to do frightening things	0.880
4. I like exciting parties	0.883
5. I would like to take off on a trip with no pre-planned routes or timetables	0.875
6. I prefer friends who are excitingly unpredictable	0.863
7. I would like to try bungee jumping	0.903
8. I would love to have new and exciting experiences, even if they are illegal	0.894
**Anxiety (M = 3.18, SD = 1.06)**	
Whenever I’m communicating with people from different cultures	
1. I feel anxious	0.821
2. I feel frustrated	0.826
3. I feel under stress	0.824
4. I feel insecure	0.823
5. I feel concerned	0.806
**Uncertainty (M = 3.22, SD = 1.01)**	
Whenever I’m communicating with people from different cultures	
1. I do not know what to expect	0.834
2. I cannot predict how the interaction would go	0.888
3. I cannot be certain on what will happen	0.867
**Mindfulness (M = 3.45, SD = 0.86)**	
1. It is easy for me to concentrate on what I am doing	0.845
2. I can tolerate emotional pain	0.842
3. I can accept things I cannot change	0.871
4. I can usually describe how I feel at the moment in considerable detail	0.838
5. I am not easily distracted	0.798
6. It’s easy for me to keep track of my thoughts and feelings	0.768
7. I try to notice my thoughts without judging them	0.816
8. I am able to accept the thoughts and feelings I have	0.808
9. I am able to focus on the present moment	0.830
10. I am able to pay close attention to one thing for a long period of time	0.809
**ICE (M = 3.46, SD = 1.10)**	
1. I communicate effectively when I engage in intercultural communication	0.901
2. My intercultural communication has always been successful	0.868
3. I feel competent when I engage in intercultural communication	0.926
4. My intercultural communication has always been a failure	0.865
5. I communicate appropriately when I engage in intercultural communication	0.868

### 3.3. Direct effects

Multivariate regression analysis was performed to examine the direct effect of all outlined predictors on ICE. The findings of this study revealed that empathy (*β* = 0.282, *t* = 3.60, *p* < 0.05), sensation seeking (*β* = 0.516, *t* = 7.93, *p* < 0.05), and mindfulness (*β* = 0.267, *t* = 3.93, *p* < 0.05) had a positive and significant influence on ICE. However, anxiety (*β* = −0.189, *t* = −3.61, *p* < 0.05) and uncertainty (*β* = −0.144, *t* = −2.51, *p* < 0.05) had a significant negative impact on ICE. Therefore, the direct effect of every individual antecedent on ICE was established and supported by the findings of this study (see Table 4).

**Table 4:** Multivariate regression analysis.

			β	S.E.	*t*	*ρ*	Status
Empathy	➔	ICE	0.282	0.078	3.601	***	Accepted
Sensation seeking	➔	ICE	0.516	0.065	7.935	***	Accepted
Anxiety	➔	ICE	−0.189	0.052	−3.611	***	Accepted
Uncertainty	➔	ICE	−0.144	0.057	−2.517	0.012	Accepted
Mindfulness	➔	ICE	0.267	0.068	3.934	***	Accepted

*** *p* < 0.001.

### 3.4. Moderating effects

In the present study, mindfulness is considered as a moderating variable, and, to establish the significant moderating effect of mindfulness, three different techniques were incorporated in this study: change in *R*^2^ values, slopes intersection, and interaction effects (46, 47). It is suggested that if the interaction term is significant then the moderating effect is established (47).

**Figure 2:**
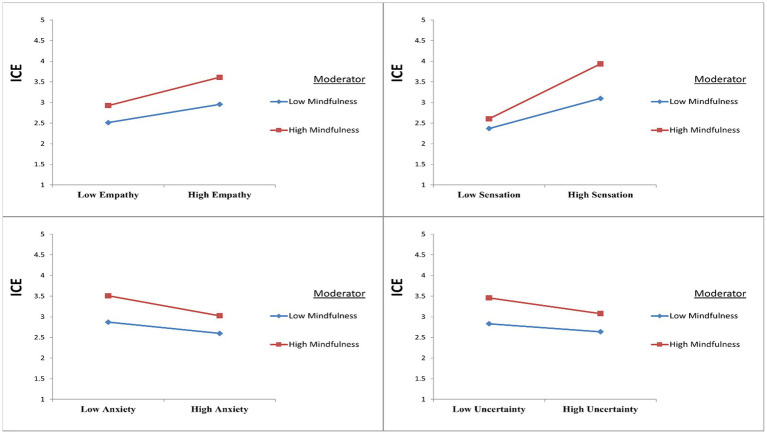
Moderating effects of mindfulness.

The study proposed that mindfulness moderates the association between empathy and ICE. The value of *R*^2^ was 0.094 before the introduction of the interaction effect (Empathy*Mindfulness). This shows that a 9.4% change in ICE is accounted for by empathy. After the inclusion of the moderating effect, the *R*^2^ value increased to 29%, showing an increase of 19.6% in variance in ICE. In addition, the interaction effect (Empathy*Mindfulness) had a statistically significant effect on ICE (*β* = 0.061, *p* < 0.05). Then, an assessment of slopes was carried out to better understand the nature of the moderating effect. Figure 2 reveals that mindfulness strengthens the positive relationship between empathy and ICE. This means that when the IS are rated at higher levels of empathy and mindfulness, they can attain more ICE.

It is proposed that mindfulness could also moderate the relationship between sensation seeking and ICE. Before the introduction of the interaction effect (Sensation Seeking*Mindfulness), the value of *R*^2^ was 0.238, showing that a 23.8% change in ICE is accounted for by sensation seeking. The *R*^2^ value increased to 31.1% after the introduction of the interaction effect, showing an increase of 7.3% in variance in ICE. Furthermore, the interaction effect (Sensation Seeking*Mindfulness) appeared to have a statistically significant effect on ICE (*β* = 0.151, *p* < 0.05). The assessment of slopes was again done to better understand the nature of the moderating effect. The pattern of slopes (see Figure 2) reveals that mindfulness strengthens the positive relationship between sensation seeking and ICE. In addition, it also confirms that higher levels of sensation seeking and mindfulness will help the IS to achieve greater ICE.

The study also proposed that mindfulness moderates the association between anxiety and ICE. The value of *R*^2^ was 0.043 before the introduction of the interaction effect (Anxiety*Mindfulness). This shows that a 4.3% change in ICE is accounted for by anxiety. After the inclusion of the moderating effect, the *R*^2^ value increased to 16.6%, showing an increase of 12.3% in variance in ICE. In addition, the interaction effect (Anxiety*Mindfulness) had a statistically significant effect on ICE (*β* = −0.053, *p* < 0.05). Again, assessment of slopes was done to better understand the nature of the moderating effect. Figure 2 reveals that mindfulness strengthens the negative relationship between anxiety and ICE. It further indicates that ICE can only be achieved by those students who have lower levels of anxiety and higher levels of mindfulness.

It is proposed that mindfulness could also moderate the relationship between uncertainty and ICE. Before the introduction of the interaction effect (Uncertainty*Mindfulness), the value of *R*^2^ was 0.004. This shows that a 0.4% change in ICE is accounted for by uncertainty. The *R*^2^ value increased to 14.8% after the introduction of the interaction effect, showing an increase of 14.4% in variance in ICE. Furthermore, the interaction effect (Uncertainty*Mindfulness) appeared to have a statistically significant effect on ICE (*β* = −0.047, *p* < 0.05). When assessment of slopes was performed to better understand the nature of the moderating effect, the pattern of slopes (see Figure 2) reveals that mindfulness strengthens the negative relationship between uncertainty and ICE. In addition, students can attain ICE when they have higher levels of mindfulness and lower levels of uncertainty. Based on the abovementioned findings, the moderating effect of mindfulness is supported by the findings of this current study.

## 4. Discussion

The current study aimed to expand the theoretical insights of AUM theory in a new cultural context (in China) under the uncertain situation engendered by several years of dealing with COVID-19. Former studies examining AUM were carried out in relatively normal or stable situations or were prone to integrate AUM into various disciplines (23–27) or to address its relevance in different cultures (18, 21, 22). As noted, no literature has been found where AUM was applied during emergency or pandemic situations. In this study, we aimed to bridge these gaps in the existing literature and to address the communication patterns of IS who stayed in China over the COVID-19 pandemic. To do so, following the trend of recent intercultural research, two additional factors (empathy and sensation seeking from IMICC) were added to expand the theoretical reach of AUM. AUM assumes that individuals need effective management of their anxiety, uncertainty, and mindfulness for the attainment of ICE or to adjust in a new culture or situation. However, the role of mindfulness on this set of factors has not been previously tested, especially on this IS population or under pandemic conditions.

In normal situations, it has been confirmed that mindfulness has favorable effects, whereas anxiety and uncertainty are adversely related to ICE. Similarly, the findings of this study revealed that this theoretical reasoning also exists under the conditions of the COVID-19 pandemic as well. The results showed that during the novel circumstances of COVID-19, the ICE of students was not deterred by anxiety and uncertainty if they found ways to apply positive factors. This indicates that their anxiety and uncertainty could be efficiently managed via ways which helped them to be effective in their intercultural interactions.

The presence of the various positive indicators such as empathy, sensation seeking, and mindfulness are also confirmed by the IS as helpfully impacting their intercultural communication, despite the negative consequences caused by COVID-19. Most importantly, even though they stayed in China through this challenging COVID-19 pandemic, their reported values suggest that they were more effective in communicating with culturally different people than might be expected. This study has shown that during COVID-19, empathy and sensation seeking had a relatively strong impact on the effective intercultural interactions of IS. The most influential moderating effect was shown to be mindfulness.

Theoretically, previous research has shown that mindfulness could help individuals to manage their levels of anxiety and uncertainty to achieve ICE (16–18). In the current study extending AUM with new factors, mindfulness was shown to further strengthen the positive influence of empathy and sensation seeking on ICE. Furthermore, mindfulness has also helped overcome the negative impact of anxiety and uncertainty on ICE. In other words, when IS have high mindfulness, they experience higher levels of empathy and sensation seeking, which will enable them to be more effective in their intercultural interactions. Furthermore, when IS attain higher levels of mindfulness, their anxiety and uncertainty will be contained to lower levels, which will lead them toward greater ICE. Mindfulness is shown to provide more positive outcomes no matter whether the affecting factors are considered positive or negative.

Going beyond the contributions noted that highlighted different cultural contexts, abnormal situations, and an extension of AUM, the current study has been validated by the individuals who belong to 42 cultural backgrounds. In early, late, and recently reported studies, AUM has indeed been validated by one or two cultural standpoints (through emic culture-specific approaches). However, this study has gained new inspirations from work on the IMICC (30, 35) and followed the culture-general approach (seeking to obtain the stance of more than two cultures, an etic across cultures) to further validate the prime assumption of AUM among IS from a wide range of cultural backgrounds. This study has reconfirmed and validated the predictions and assumptions of AUM in the midst of the pandemic in the unique cultural setting of China.

Therefore, this integration of AUM with IMICC factors has provided new insights to understand the communication of IS during the COVID-19 pandemic. It can be argued that, despite numerous psychological, situational, and contextual challenges, IS that stayed in China found ways to be strong enough to deal with the COVID-19 pandemic, and furthermore, these challenges did not generally adversely influence their intercultural communication. The results of this study are consistent with the claims of AUM that ICE is achieved by managing anxiety and uncertainty. IMICC predictions regarding empathy and sensation seeking are also supported by this study’s findings in that these two factors enable people to be competent in intercultural communication. Linking the two together has provided a broader explanatory model for moderating effects, particularly identifying how mindfulness in particular moderates both of AUM’s negative conditions and IMICC’s positive pathways.

Practically, the findings of this research could benefit China in different ways. First, with the sharp decline of IS going to China observed during the COVID-19 pandemic, the Chinese Ministry of Education could consider reframing their existing policies to provide better support for IS who are planning to pursue their higher education in China, as the country has again opened its borders for such students. Second, host institutes could adopt the IMICC-modified AUM framework used in this research and could accordingly conduct training sessions for newly arrived students to further strengthen these factors (variables of this study) to make them effective in intercultural communication during normal conditions (considering the post-COVID-19 scenarios). Third, the arriving IS could take the initiative and participate in improving their own intercultural interactions just like the participants of this research have done. Moreover, they can consider ways of developing mindfulness to help them better manage the other factors. All these proposed initiatives have the potential to eventually bring good results for China in its goals to become a major Asian and global hub recruiting and developing IS.

To sum up, it is observed that COVID-19 pandemic–related investigations in China have documented the psychological conditions of healthcare workers, postgraduate students, mothers, children, and adolescents, as well as patients with the infection, recovered from the virus, and visiting psychiatric departments (48–54). On the other hand, psychological factors influencing the intercultural communication of IS have remained unnoticed. The findings of this current research have confirmed that the psychological condition of IS, in terms of their intercultural interactions in China during the COVID-19 pandemic, was stable and effective compared to other segments of the Chinese population.

### 4.1. Limitations

Regarding limitations, the study is confined to the cultural context of China in the abnormal context of the COVID-19 pandemic period. The sample is also smaller than desired due to the restricted population of those IS who stayed in China throughout the nearly 3 years (2020–2022) up to the end of the pandemic measures. Determining any cause-and-effect relations could not be established due to the cross-sectional research approach. The results may also be subjected to social desirability biases common in self-report measures. The current study is limited to various hidden latent variables, such as situational factors, personality, and contextual influences, that are not directly considered in this research, which might have affected the findings. Therefore, future research should explore these types of factors in examining the ICE of individuals. Domestic or regional student populations also remained unexplored in this study. Future research can focus on how they dealt with and processed AUM and ICE factors during COVID-19, which could also yield insights for the further development of these theories and their applications.

## 5. Conclusion

The aim of this study was to extend the theoretical predictions of AUM theory during the COVID-19 pandemic among IS in China to inspect factors affecting their communication effectiveness during the lockdown. The findings provide evidence that the inclusion of empathy and sensation seeking has contributed explanatory power to the theoretical insights of AUM. Despite the intensity of the COVID-19 pandemic, IS remaining in China have somehow managed their anxiety and uncertainty levels, remaining mindful while interacting with culturally different people, and reported that the traits of empathy and sensation seeking also enabled them to achieve ICE. Thus, the results confirm the prediction that AUM still holds true whether in normal or more stressful pandemic situations. For future studies, further comparisons among different counties during this pandemic and beyond could further enhance insights into the scope and explanatory power of AUM theory and the psychological dispositions that best moderate effective intercultural communication.

## Data availability statement

The raw data supporting the conclusions of this article will be made available by the authors, without undue reservation.

## Ethics statement

The studies involving human participants were reviewed and approved by Institutional Ethics Review Committee of the SISU Intercultural Institute (SII), Shanghai International Studies University (SISU), China (2023-SII/IRB-1017). The patients/participants provided their written informed consent to participate in this study.

## Author contributions

MN: investigation, conceptualization, methodology, validation, data collection, formal analysis, and writing – original draft preparation. SK: conceptualization, methodology, resources, and writing – review and editing. AZ: conceptualization, formal analysis, and writing – review and editing. IB: conceptualization, formal analysis, and writing – review and editing. All authors contributed to the article and approved the submitted version.

## Funding

This study was conducted as an extended part of the (post-doctoral) research program of the SISU Intercultural Institute, Shanghai International Studies University (SISU), Shanghai, China, related to the 2021–2024 SISU University Key Project Number 2021114007 (studies on the mechanisms of intercultural communication and interactions in international communication channels).

## Conflict of interest

The authors declare that the research was conducted in the absence of any commercial or financial relationships that could be construed as a potential conflict of interest.

## Publisher’s note

All claims expressed in this article are solely those of the authors and do not necessarily represent those of their affiliated organizations, or those of the publisher, the editors and the reviewers. Any product that may be evaluated in this article, or claim that may be made by its manufacturer, is not guaranteed or endorsed by the publisher.
